# Identifying Nursing-Sensitive Indicators for Hospitals: A Modified Delphi Approach

**DOI:** 10.7759/cureus.59472

**Published:** 2024-05-01

**Authors:** Tareq Afaneh, Fathieh Abu-Moghli, Maha Mihdawi

**Affiliations:** 1 Nursing, Bahrain Oncology Center, Al Muharraq, BHR; 2 Community Health Nursing, School of Nursing, Jordan University, Amman, JOR; 3 Nursing Research, King Hamad University Hospital, Al Muharraq, BHR

**Keywords:** delphi method, quality of healthcare, quality indicators, practice, nurse sensitive indicators, nurses

## Abstract

Background: Nursing-sensitive indicators (NSIs) play a crucial role in measuring the quality of care specific to nursing practice. Currently, hospitals monitor several NSIs which may vary between hospitals. Conducting research on NSIs can enhance the monitoring of nursing practice.

Aim: The aim is to identify NSIs for hospitals in Jordan.

Methods and material: The Delphi approach was utilized to establish a consensus among a panel of national nursing experts (N=60). An initial list of 52 indicators was developed through a rigorous process and subsequently distributed to the panel members. The panelists provided their quantitative responses in three rounds. Consensus was determined based on the following criteria: agreement greater than 51.0%, interquartile range (IQR) below 1.5, standard deviation (SD) below 1, and moderate Kendall’s coefficient of concordance (Kendall’s W).

Results: By the conclusion of the third round, a total of 42 indicators achieved group agreement. The agreed-upon indicators consisted of 10 structure, 16 process, and 16 outcome indicators.

Conclusion: This study successfully established a consensus and identified a comprehensive set of indicators that capture the distinct contributions of nursing in the hospital setting. The results demonstrate a wide range of agreed-upon indicators across the domains of structure, process, and outcome. These findings are valuable in enhancing the monitoring and evaluation of nursing practice in hospitals.

Practical implications: The findings of this study provide a solid foundation for monitoring and reporting the quality of nursing practice in hospitals. Nursing policymakers can utilize these findings to develop policies that promote the voluntary reporting of NSIs.

## Introduction

The importance of assessing the quality of nursing care and the need for comprehensive measurement models have been recognized in healthcare. A seminal contribution in this regard is the classic work of Donabedian [1], who provided a comprehensive and conceptually based model to measure different aspects of the healthcare system. This model emphasized the measurement of structure, process, and outcome indicators. Building upon this foundation, Dubois et al. adopted Donabedian's framework to measure aspects of nursing performance. According to Dubois, nursing performance can be understood as the outcome of the interaction between three subsystems. The first subsystem involves acquiring, deploying, and maintaining nursing resources. It is defined as the attributes of the nursing system that affect its ability to meet healthcare needs, such as nursing staff attributes and the stability of financial resources. The second subsystem is transforming nursing resources into nursing services, which includes nurses’ activities and the attributes of the practice environment, such as nursing processes. The third subsystem is producing changes in patients’ condition, encompassing the health status or events resulting from nursing care, such as patient safety outcomes, patient functional status, and patient length of stay [2].

Gao et al. [3] used Heslop et al.'s [4] definition of nursing-sensitive quality indicators as a set of principles, procedures, and assessment scales used to quantify the level of nursing quality and assess nursing outcomes in clinical nursing practice. Kieft et al. [5] also employed Heslop et al.'s definition of nurse-sensitive indicators as quantifiable items that monitor or provide an indication of the quality of the nursing care provided. McNett [6] defined nursing-sensitive indicators (NSIs) as measurement indices that reflect the quality of nursing care provided in a unit or a hospital. Stalpers et al. [7] defined nurse-sensitive indicators as outcomes that are relevant, based on nurses’ scope of practice, and for which there is empirical evidence linking nursing inputs and interventions to the outcome of patient care.

NSIs serve as a tool for systemically assessing the quality of nursing care [5,8]. Monitoring NSIs can identify significant areas for improving nursing care quality and allow the focus of improvement efforts on priority areas [9].

The availability of a national database that provides periodic reporting of structure, process, and outcome NSIs allows for conducting benchmarks with countries of similar socioeconomic development. Furthermore, NSIs provide measurable evidence for nursing policymaking by establishing linkages between nursing resources, such as nurse staffing levels and nursing levels of education, and patient outcomes. In light of these considerations, the present study aims to identify NSIs for hospital use.

## Materials and methods

Design, setting, and participants

The present study employed the Delphi method. The initial description of the Delphi method appeared in the 1950s by RAND Air Force Corporation to predict how technology impacts warfare [10]. The method has lent itself to nursing and health research for more than three decades. Early applications of the method included deciding on future research priorities. Later, the applications of the method expanded to include deciding on policy-making and analysis options, assessing the healthcare outcomes, and determining the relevance of practice to health-related outcomes [11,12].

The study was conducted in public and private hospitals in Jordan. The healthcare system in Jordan includes both private and public hospitals. The public sector hospitals include the Ministry of Health (MOH) Hospitals, the Royal Medical Services (RMS) hospitals, and University Hospitals. The expert panel was recruited from nurses holding three managerial positions: Directors of Nursing (DONs), nursing shift supervisors, and nursing quality specialists. There is no general rule for determining the required number of experts in the Delphi method, as the number of experts varies [13]. The sample size in Delphi ranges from four to 3,000 participants, with 10 to 100 participants in most healthcare-related studies. The number is usually determined based on pragmatic considerations [13,14]. The more intellectual diversity invested in a study, the more knowledge is expected to be generated. Yet, a larger number of experts is linked to administrative inefficiencies, such as the need for more financial resources and time. The type of required information is another factor; in-depth information is best achieved by a relatively smaller number of experts than structured information [15]. This study sought to recruit 40-60 participants who meet the inclusion criteria: holding a nursing bachelor’s degree as a minimum qualification; ideally holding a postgraduate qualification in nursing; currently working in a managerial position as DON, nursing supervisor, or nursing quality specialist, having more than five years of working experience, and willingness to invest six to 10 weeks in participating in the study. A purposive sampling method was used to recruit the participants. Participants who met these selection criteria were identified as possessing the required expertise to contribute to the study.

Data collection

To develop the initial list of NSIs, a search was performed using PubMed, MEDLINE, and CINAHL full text in the EBSCOhost databases. The subject headings “nurse* sensitive indicators” and “nurse* sensitive outcomes” were entered. The search included full-text articles published in English from 2009 to 2020. Eligible sources incorporated articles from peer-reviewed journals with any study design.

The first-round survey included three parts: i) study information and instructions, ii) the demographic characteristics of the panelists, and iii) a list of potential NSIs that was developed by the authors after reviewing pertinent literature [16]. The order of the identified NSIs in the instrument was based conceptual definition of the three subsystems of the Nursing Care Performance Framework (NCPF) [2]. This process resulted in categorizing NSIs into three sections resembling the three subsystems of the NCPF. A five-point Likert scale ranging from “strongly disagree” to “strongly agree” was implemented to score the level of agreement.

The authors sent invitation letters to each hospital inviting the DON, nursing supervisors, and nursing quality specialists to participate in the study. The total number of hospitals that were contacted is 24 hospitals, and 19 hospitals responded. The study included multiple rounds. Each round of the study was accessible for a duration of two weeks. The time interval between the closure of one round and the opening of the next round was two weeks.

In round 1, the participants were offered a list of 52 NSIs by individual emails and were asked to suggest additional NSIs. Potential NSIs resulting from the literature review and participants' input were utilized to develop the study questionnaire and were included in the subsequent rounds.

During round 2, each participant was asked to determine their level of agreement on a five-point Likert scale as follows: “strongly disagree” (1), “disagree” (2), “neutral” (3), “agree” (4), and “strongly agree” (5). The item agreement index is defined as achieving at least 51% of responses as “agree” or “strongly agree,” an Interquartile Range (IQR) below 1.5, and a Standard Deviation (SD) below 1 [13].

**Figure 1:**
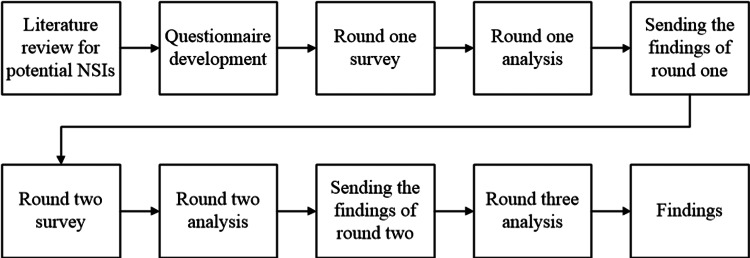
Summary of data collection procedure

Two weeks after the closure of round 2, the third round was released. During this round, the participants received individual feedback on the group response findings of round two through their email addresses to maintain anonymity. This feedback allowed the participants to assess the group response to each NSI. The participants were asked to reevaluate all the indicators considering the findings of round 2. A summary of data collection is presented in Figure 1.

Data analysis

Participants' responses were transferred to SPSS Statistics 22.0 for Windows (IBM Corp., Armonk, NY) [17]. Mean, median, frequency, IQR, and SD were calculated for each indicator. Responses of “agree” and “strongly agree” for each indicator were combined to form “agreed.” The percentage of agreed responses for each indicator was calculated. Indicators that met the three identified criteria (51% or greater of agreed responses, IQR below 1.5, and SD below 1) in the final round are identified as agreement indicators [13]. The overall agreement among experts was estimated using Kendall’s coefficient of concordance (Kendall’s W).

Ethical considerations

Institutional review board (IRB) approval was secured prior to contacting the participants from each hospital. Confidentiality of the responses was assured to the participants, and their responses were kept confidential. Ethical considerations were explained in the consent form that was obtained from the participants.

## Results

Demographic characteristics

The panel members' ages ranged from 25 and 62 years (M = 40.63, SD = 7.50). The participants included full-time quality specialists (n=24, 40%), nursing supervisors (n=21, 35%), and directors of nursing (n = 15, 25%). Their total years of clinical experience ranged from 6 -36 years (M=18.47, SD=7.10). The majority of panel members (n = 37, 61.7%) held a bachelor’s degree, followed by participants with a master’s degree (n=20, 33.3%), and a PhD (n=3, 5.0%). A summary of the demographic characteristics is presented in Table 1.

**Table 1:** Participants’ characteristics N= (60)

	N (%)	Min	Max	Mean (SD)	Median
Age		25	62	40.63 (7.50)	40.50
Current position					
Director of Nursing	15 (25)				
Nursing Supervisor	21 (35)				
Quality Specialist	24 (40)				
Total Experience (years)		6	36	18.47 (7.10)	18.5
Academic Qualification					
Doctorate	3 (5.0)				
Master	20 (33.3)				
Bachelor	37 (61.7)				
Hospital Sector					
Public	24 (40)				
Private Hospital	36 (60)				
Hospital Bed Capacity		42	678	266.27(157.84)	200

Round 1

The literature review and the input from the participants resulted in the identification of 52 indicators for inclusion in the subsequent rounds of the study. No additional NSIs were suggested by the participants. The study encompassed 20 process indicators, 19 outcome indicators, and 13 structure indicators. All the identified indicators were included in the study. The indicators resulting from round 1 are listed in Table 2.

**Table 2:** Initial list of indicators

Indicator
Structure indicators
Working conditions (Employment conditions, Stability, Workload)
Staff maintenance (Retention/turnover)
Nursing staff supply (Quantity/intensity)
Nurse-Bed ratio
Staff maintenance (Absenteeism)
Staff maintenance (Work-related accidents, injuries, illnesses)
Nursing staff supply (Quality/training/experience)
Working conditions (Support resources, Physical facilities, Material resources)
Staff maintenance (Satisfaction at work)
Working Hours Per Patient Days
Nursing staff supply (Patient classification) systems
Economic sustainability (Cost per case-mix or patient-day)
Economic sustainability (Cost of resources)
Process indicators
Communication (Nurse-Patient)
Professional satisfaction
Deployment of scope of practice
Pain assessment
Nursing work environment characteristics (perceived autonomy, role tension, collaboration)
Job burnout
Collaboration (Nurse-Patient)
Discharge planning
Patient centrality in the nursing care delivery process (Patient/family involvement)
Nursing processes
Inter Unit Work Relations
Patient centrality in the nursing care delivery process (Responsiveness to patients’ needs and expectations)
Restraint application
Health Promotion and Illness Prevention
Problems & symptoms management
Patient centrality in the nursing care delivery process (Continuity, reactivity, timeliness, coordination)
Malnutrition screening
Nurse decision making
Conflict resolution (Nurse-Patient)
Delirium observation
Outcome indicators
Patient Fall
Patient satisfaction
Central line infection rates (Central Line-Associated Bloodstream Infections)
Catheter Associated Urinary Tract Infection
Intra-venous infection (phlebitis)
Patient comfort and quality of life related to care: Hygiene
Hospital acquired Infection
Pressure Injury
Hospital Acquired Pneumonia
Failure to rescue
Patient empowerment: Ability to achieve appropriate self-care
Patient comfort and quality of life related to care: Symptoms management (e.g., pain, nausea, dyspnea, fever)
Medication error
Patient comfort and quality of life related to care: Incontinence
Patient functional status (physical, nutritional)
Patient empowerment: Adoption of health-promoting behaviors
Average Length of Stay
Mortality rate
Re-admissions

Round 2

Out of the 52 items entered in round 1, 36 items met the criteria for agreement. The percentages of agreement ranged from 51% (delirium observation and malnutrition screening) to 97.5% (nurse-patient communication). The indicators that survived round 2 of the study comprised five structure indicators, 15 process indicators, and 16 outcome indicators. However, there were eight structure indicators, five process indicators, and three outcome indicators that did not meet the agreement criteria by the end of round 2.

The overall agreement between experts was estimated using Kendall’s coefficient of concordance (Kendall’s W), which is a non-parametric statistic used to assess agreement among raters. Kendall's W ranges from zero (no agreement) to one (complete agreement). For round 2 responses, Kendall’s W was found to be .13, indicating poor agreement among the experts. Based on this finding, the decision was made to proceed to round 3. These indicators are provided in Table 3.

**Table 3:** Indicators that achieved agreement by the end of round 2 *Indicators that achieved agreement criteria

Indicator	Mean	Median	SD	IR	% of agreement
Structure indicators					
Nursing staff supply (Quality/training/experience)*	4.33	5	0.95	1	88%
Staff maintenance (Satisfaction at work)	4.22	4	1.03	1	88%
Staff maintenance (Retention/turnover) *	4.18	4	0.97	1	88%
Working conditions (Employment conditions, Stability, Workload) *	4.18	4	0.83	1	87%
Nursing staff supply (Quantity/intensity)	3.98	4	1.1	1	83%
Staff maintenance (Work-related accidents, injuries, illnesses) *	4.03	4	0.9	1	82%
Working Hours Per Patient Days	3.78	4	1.12	0	80%
Nurse-Bed ratio	4.12	4.5	1.17	1	80%
Nursing staff supply (Patient classification) systems	3.82	4	1.03	0	78%
Working conditions(Support resources, Physical facilities, Material resources)	3.85	4	1.15	1	78%
Staff maintenance (Absenteeism)*	3.87	4	0.98	1	77%
Economic sustainability (Cost per case-mix or patient-day)	3.58	4	1.03	1	68%
Economic sustainability (Cost of resources)	3.87	4	1.13	1	57%
Process indicators					
Communication (Nurse-Patient) *	4.5	5	0.62	1	97%
Patient centrality in the nursing care delivery process (Patient/family involvement) *	4.18	4	0.7	1	93%
Collaboration (Nurse-Patient) *	4.28	4	0.78	1	92%
Patient centrality in the nursing care delivery process (Continuity, reactivity, timeliness, coordination) *	4.15	4	0.66	1	92%
Pain assessment*	4.35	5	0.82	1	88%
Nursing processes*	4.22	4	0.74	1	88%
Patient centrality in the nursing care delivery process (Responsiveness to patients’ needs and expectations) *	4.07	4	0.71	0	88%
Inter Unit Work Relations*	4.1	4	0.8	1	87%
Deployment of scope of practice*	4.12	4	0.87	1	85%
Professional satisfaction*	4.12	4	0.78	1	85%
Discharge planning*	3.95	4	0.93	1	83%
Nurse decision making	4.08	4	1.03	1	82%
Conflict resolution (Nurse-Patient	4	4	1.03	1	80%
Nursing work environment characteristics (perceived autonomy, role tension, collaboration) *	3.82	4	0.95	0	80%
Restraint application*	3.88	4	0.89	0	78%
Health Promotion and Illness Prevention*	3.88	4	0.87	0	77%
Job burnout*	3.88	4	0.9	0	77%
Problems & symptoms management	3.88	4	0.96	2	75%
Delirium observation	3.28	3.5	1.03	1	50%
Malnutrition screen	3.23	3.5	1.06	2	51%
Outcome indicators					
Patient comfort and quality of life related to care: Hygiene*	4.3	4	0.67	1	95%
Pressure Injury*	4.3	4	0.79	1	92%
Patient Fall*	4.43	5	0.85	1	92%
Patient comfort and quality of life related to care: Symptoms management (e.g., pain, nausea, dyspnea, fever) *	4.15	4	0.78	1	92%
Intra-venous infection (phlebitis)*	4.33	4	0.8	1	90%
Patient satisfaction*	4.15	4	0.8	1	88%
Medication error*	4.3	5	0.94	1	88%
Central line infection rates (Central Line-Associated Bloodstream Infections) *	4.25	4	0.93	1	87%
Catheter Associated Urinary Tract Infection*	4.13	4	0.89	1	87%
Hospital Acquired Pneumonia*	4.05	4	0.91	1	85%
Hospital acquired Infection*	4.22	4	0.83	1	85%
Failure to rescue*	3.78	4	0.96	1	75%
Patient empowerment: Ability to achieve appropriate self-care*	3.8	4	0.84	1	75%
Patient comfort and quality of life related to care: Incontinence*	3.73	4	0.9	1	72%
Patient empowerment: Adoption of health-promoting behaviors*	3.73	4	0.9	1	72%
Patient functional status (physical, nutritional) *	3.63	4	0.88	1	72%
Average Length of Stay	3.67	4	1.13	1	68%
Re-admissions	3.53	4	1.21	2	65%
Mortality rate	3.38	4	1.15	2	55%

Round 3

All indicators that met the agreement in round 2 (36 indicators) were maintained in round 3. Additionally, six indicators were retained from the 16 dropped indicators in round 2. These retained indicators include working hours per patient days, nursing staff supply (quantity/intensity), working conditions (support resources, physical facilities, material resources, staff maintenance), satisfaction at work, nurse-bed ratio, and problems and symptoms management. As a result, there were 42 indicators categorized as follows: 10 structure indicators, 16 process indicators, and 16 outcome indicators. The percentages of agreement ranged from 11% (patient re-admissions) to 100% (Nurse-Patient Communication, Professional satisfaction, Patients Falls, Patient satisfaction, Central line infection rates, Catheter-Associated Urinary Tract Infection, Intra-venous infection, Patient comfort and quality of life-related, and Hospital-acquired Infection).

The overall agreement between experts was estimated using Kendall’s coefficient of concordance (Kendall’s W). For round 3 responses, Kendall’s W was found to be 0.41, indicating moderate agreement between the experts [18]. Based on these findings, a decision was made to conclude the study at the end of round three for the following reasons: First, there was stability of responses between round 2 and round 3, as most of the agreed-upon indicators remained consistent in the two rounds (36/42). Second, a moderate level of agreement was achieved between experts. Third, the participants showed signs of fatigue, as the dropout rate increased from 8% in round 2 to 15% in round 3. It was expected that the dropout rate would further increase if the study proceeded to round 4. A summary of the results is presented in Table 4.

**Table 4:** Participants responses in round 3. * Indicators that achieved agreement criteria Ø Indicators that did not achieve agreement criteria

Indicator	Mean	Median	SD	IR	% of agreement
Structure indicators					
Working conditions (Employment conditions, Stability, Workload) *	4.11	4	0.37	0	98
Staff maintenance (Retention/turnover) *	4.27	4	0.49	1	98
Nursing staff supply (Quantity/intensity) *	4.13	4	0.47	0	95
Nurse-Bed ratio*	4.27	4	0.49	1	95
Staff maintenance (Absenteeism)*	4.02	4	0.41	0	93
Staff maintenance (Work-related accidents, injuries, illnesses) *	4.13	4	0.55	0	91
Nursing staff supply (Quality/training/experience*	4.33	4	0.67	1	89
Working conditions (Support resources, Physical facilities, Material resources) *	3.91	4	0.7	0	82
Staff maintenance (Satisfaction at work) *	3.78	4	0.83	1	71
Working Hours Per Patient Days*	3.47	4	0.69	1	58
Nursing staff supply (Patient classification) systems Ø	3.04	3	0.82	2	35
Economic sustainability (Cost per case-mix or patient-day) Ø	2.96	3	0.86	2	35
Economic sustainability (Cost of resources) Ø	2.87	3	0.7	1	18
Process indicators					
Communication (Nurse-Patient) *	4.6	5	0.49	1	100
Professional satisfaction*	4.31	4	0.47	1	100
Deployment of scope of practice*	4.13	4	0.34	0	99
Pain assessment*	4.33	4	0.51	1	98
Nursing work environment characteristics (perceived autonomy, role tension, collaboration) *	4.24	4	0.47	1	98
Job burnout*	4.11	4	0.37	0	98
Collaboration (Nurse-Patient) *	4.25	4	0.55	1	95
Discharge planning*	4.11	4	0.6	0	95
Patient centrality in the nursing care delivery process (Patient/family involvement) *	4.02	4	0.41	0	93
Nursing processes*	3.96	4	0.47	0	91
Inter Unit Work Relations*	4.18	4	0.77	1	89
Patient centrality in the nursing care delivery process (Responsiveness to patients’ needs and expectations) *	4.02	4	0.49	0	89
Restraint application*	3.93	4	0.42	0	87
Health Promotion and Illness Prevention*	3.91	4	0.44	0	85
Problems & symptoms management*	3.85	4	0.49	0	80
Patient centrality in the nursing care delivery process (Continuity, reactivity, timeliness, coordination) *	3.87	4	0.64	0	76
Malnutrition screening Ø	3.29	4	0.81	1	51
Nurse decision making Ø	3.25	3	0.75	1	44
Conflict resolution (Nurse-Patient) Ø	2.93	3	0.92	2	35
Delirium observation Ø	3.07	3	0.57	0	20
Outcome indicators					
Patient Fall*	4.45	4	0.5	1	100
Patient satisfaction*	4.31	4	0.47	1	100
Central line infection rates (Central Line-Associated Bloodstream Infections) *	4.36	4	0.49	1	100
Catheter Associated Urinary Tract Infection*	4.31	4	0.47	1	100
Intra-venous infection (phlebitis)*	4.36	4	0.49	1	100
Patient comfort and quality of life related to care: Hygiene*	4.09	4	0.29	0	100
Hospital acquired Infection*	4.15	4	0.36	0	100
Pressure Injury*	4.22	4	0.46	0	98
Hospital Acquired Pneumonia*	4.24	4	0.47	1	98
Failure to rescue*	4	4	0.27	0	96
Patient empowerment: Ability to achieve appropriate self-care*	4.07	4	0.47	0	96
Patient comfort and quality of life related to care: Symptoms management (e.g., pain, nausea, dyspnea, fever) *	4.04	4	0.47	0	95
Medication error*	4.25	4	0.64	1	93
Patient comfort and quality of life related to care: Incontinence*	4	4	0.38	0	93
Patient functional status (physical, nutritional) *	3.8	4	0.62	0	84
Patient empowerment: Adoption of health-promoting behaviors*	3.96	4	0.67	0	75
Average Length of Stay Ø	3.29	3	0.74	1	45
Mortality rate Ø	3.16	3	0.74	1	36
Re-admissions Ø	2.82	3	0.61	1	11

## Discussion

The present study's agreed-upon NSIs in the “structure” category align with commonly reported structure indicators in the literature. Previous studies by Burston et al. and Xu identified nurse-patient ratio, Nursing Hours Per Patient Day (NHPPD), education, and years of experience as commonly used structure NSIs [19,20]. Another study conducted in Iran also highlighted nurses’ education and experience, nurse-to-patient ratio, and the ratio of Registered Nurses to the nursing staff as important structure indicators [21]. However, none of the structure NSIs related to the economic stability of the nursing system achieved agreement in this study, which may be attributed to insufficient educational preparation for nurses on fiscal management and nursing economics [22,23].

The literature describes the relationship between the structure components and the quality of nursing performance for some NSIs. For example, Kim and Bae conducted a retrospective observational study on 338,369 patients and found significant relationships between nurse staffing levels and six nursing-sensitive outcomes [24]. Kouatly et al. [25] assessed the association between nurse staffing levels and patient outcomes or nursing-sensitive outcomes in in-patient units and found that lower working hours per patient day were significantly associated with falls, injury falls, and hospital-acquired pressure injuries as well as central line-associated bloodstream infection (CLABSI) in medical-surgical units. Further research studies can be conducted to support the association between structural components of the nursing system and patients' outcomes, which can inform evidence-based decision-making regarding health policy and the distribution of nursing resources.

Unlike structure indicators, process indicators require active monitoring using various methods. Process indicators often have limited evidence linking them to outcome indicators [4]. However, the impact of process Indicators on quality of care should not be underestimated. A study by Rastian et al. revealed a discrepancy between the quality of care before and after implementing and mentoring the nursing process [26].

The present study found that more than one-third of the agreed-upon NSIs were outcome indicators (16 out of 42). This finding is consistent with the understanding of the nurses' primary responsibility in producing positive patient care outcomes. It also aligns with international findings on outcome indicators [2,19,20].

The NCPF identifies five areas as joint outcome contributions by nursing and other systems: health status, readmission, length of stay, complications, and mortality. However, in this study, these areas did not achieve agreement as Nursing-Sensitive Indicators, as reflect higher-level organization-wide key performance indicators (KPIs) rather than NSIs [27]. The findings of the present study can support the national strategy for nursing and midwifery by developing an agreed-upon national quality nursing indicators dataset. Monitoring NSIs helps analyze the impact of nursing performance on patient healthcare outcomes, provides knowledge to implement evidence-based nursing improvements, and convinces healthcare policymakers of the impact of nursing practice on health outcomes.

Both mortality rate and readmission rate are considered outcomes for joint contribution by different health care disciplines. Despite nursing care can affect both mortality and readmission, these two outcomes are not specific to nursing care and may represent hospital-level KPIs rather than NSIs. Perhaps this is why they generated low agreement among the participants to be considered NSIs [2].

Despite contributing to the breadth of knowledge about NSIs, the present study has some limitations inherent in the Delphi method, such as the lack of an agreed statistical cutoff point for agreement among participants, the absence of criteria for defining experts, and potential validity issues with participants' responses in the Delphi technique.

Implications for international practice

Healthcare and nursing policymakers can adopt and implement a nationwide policy for the voluntary and mandatory monitoring and reporting of the agreed-upon structure, process, and outcome NSIs. These Indicators can be utilized to measure and benchmark the quality and performance of nursing care at the national levels, as well as to make comparisons with countries of similar socio-economic status. The adoption and reporting of this policy can serve broader goals beyond benchmarking healthcare organizations for improvement. It can also provide evidence of the efficiency of the nursing system by comparing available resources with patient care outcomes.

Continued nursing research on NSIs is imperative for several reasons. Firstly, measuring quality is a prerequisite for improving it. Secondly, nursing is a profession with a unique scope of practice, necessitating a distinctive system for monitoring the quality of provided by nurses. Thirdly, nursing leaders and policymakers require a valid and reliable tool to measure the quality of nursing care. Lastly, further evidence is needed to strengthen the position of nursing policymakers in advocating for investment in the nursing workforce.

## Conclusions

This study represents an endeavor to enhance the clarity of NSIs by proposing a scientifically and conceptually guided agreement on these indicators. Achieving clarity in NSIs is essential to advance the quantification of the quality of nursing care. NSIs also contribute to researchers’ efforts in providing robust evidence regarding the impact of the nursing profession on patient healthcare outcomes. The adoption and implementation of a national database for both mandatory and voluntary reporting of the agreed-upon structure, process, and outcome of NSIs would provide nursing policymakers with a reliable tool to support decision-making.

## References

[1] Donabedian A (1988). The quality of care: how can it be assessed?. JAMA.

[2] Dubois CA, D'Amour D, Pomey MP, Girard F, Brault I (2013). Conceptualizing performance of nursing care as a prerequisite for better measurement: a systematic and interpretive review. BMC Nurs.

[3] Gao JL, Liu XM, Che WF, Xin X (2018). Construction of nursing-sensitive quality indicators for haemodialysis using Delphi method. J Clin Nurs.

[4] Heslop L, Lu S, Xu X (2014). Nursing-sensitive indicators: a concept analysis. J Adv Nurs.

[5] Kieft RA, Stalpers D, Jansen AP, Francke AL, Delnoij DM (2018). The methodological quality of nurse-sensitive indicators in Dutch hospitals: a descriptive exploratory research study. Health Policy.

[6] McNett M (2018). Nursing-sensitive outcomes after severe traumatic brain injury: a nationwide study. J Neurosci Nurs.

[7] Stalpers D, Kieft RA, van der Linden D, Kaljouw MJ, Schuurmans MJ (2016). Concordance between nurse-reported quality of care and quality of care as publicly reported by nurse-sensitive indicators. BMC Health Serv Res.

[8] Afaneh T, Abu-Moghli F, Ahmad M (2021). Nursing-sensitive indicators: a concept analysis. Nurs Manag (Harrow).

[9] Sim J, Joyce-McCoach J, Gordon R, Kobel C (2019). Development of a data registry to evaluate the quality and safety of nursing practice. J Adv Nurs.

[10] Arakawa N, Bader LR (2022). Consensus development methods: considerations for national and global frameworks and policy development. Res Social Adm Pharm.

[11] Waltz CF, Strickland OL, Lenz ER (2016). Measurement in Nursing and Health.

[12] Olsen AA, Wolcott MD, Haines ST, Janke KK, McLaughlin JE (2021). How to use the Delphi method to aid in decision making and build consensus in pharmacy education. Curr Pharm Teach Learn.

[13] Giannarou L, Zervas E (2014). Using Delphi technique to build consensus in practice. Int J Business Sci Appl Manag.

[14] Thangaratinam S, Redman CW (2005). The Delphi technique. Obstetr Gynaecol.

[15] Rowe G, Wright G (2001). Expert opinions in forecasting: the role of the Delphi technique. International Series in Operations Research & Management Science.

[16] Afaneh T, Abu-Moghli FA (2020). Consistency of nursing directors, nursing supervisors, and nursing quality specialists’ perception about nursing-sensitive indicators in acute care settings. Open J Nurs.

[17] (2024). IBM SPSS Statistics for Windows. https://www.ibm.com/support/pages/how-cite-ibm-spss-statistics-or-earlier-versions-spss.

[18] Landis JR, Koch GG (1977). The measurement of observer agreement for categorical data. Biometrics.

[19] Burston S, Chaboyer W, Gillespie B (2014). Nurse-sensitive indicators suitable to reflect nursing care quality: a review and discussion of issues. J Clin Nurs.

[20] Xu X (2015). Identification of nursing-sensitive indicators for nursing quality monitoring and reporting in an Australian context. Thesis Doctor of Philosophy. Victoria University.

[21] Oner B, Zengul FD, Oner N, Ivankova NV, Karadag A, Patrician PA (2021). Nursing-sensitive indicators for nursing care: a systematic review (1997-2017). Nurs Open.

[22] Bai Y, Gu C, Chen Q, Xiao J, Liu D, Tang S (2017). The challenges that head nurses confront on financial management today: a qualitative study. Int J Nurs Sci.

[23] Arnaert A, Mills J, Bruno FS, Ponzoni N (2018). The educational gaps of nurses in entrepreneurial roles: an integrative review. J Prof Nurs.

[24] Kim CG, Bae KS (2018). Relationship between nurse staffing level and adult nursing-sensitive outcomes in tertiary hospitals of Korea: Retrospective observational study. Int J Nurs Stud.

[25] Kouatly IA, Nassar N, Nizam M, Badr LK (2018). Evidence on nurse staffing ratios and patient outcomes in a low-income country: implications for future research and practice. Worldviews Evid Based Nurs.

[26] Rastian ML, Sadeghi F, Sadeghi N (2016). Effect of implementing nursing process on the quality of patient care in surgical wards. Int J Adv Biotechnol Res.

[27] Dubois CA, D'amour D, Brault I (2017). Which priority indicators to use to evaluate nursing care performance? A discussion paper. J Adv Nurs.

